# Odor-Induced Neuronal Rhythms in the Olfactory Bulb Are Profoundly Modified in ob/ob Obese Mice

**DOI:** 10.3389/fphys.2017.00002

**Published:** 2017-01-19

**Authors:** Yan Chelminski, Christophe Magnan, Serge H. Luquet, Amandine Everard, Nicolas Meunier, Hirac Gurden, Claire Martin

**Affiliations:** ^1^UMR 8165 Centre National de la Recherche Scientifique, IMNC, Paris Sud University, Paris Diderot UniversityOrsay, France; ^2^UMR 8251 Centre National de la Recherche Scientifique, BFA, Paris Diderot University, Sorbonne Paris Cité UniversityParis, France; ^3^INRA, UR1197 NeuroBiologie de l'OlfactionJouy-en-Josas, France; ^4^Université de Versailles St-Quentin en YvelinesVersailles, France

**Keywords:** leptin, ob/ob mice, odor-induced oscillations, learning, olfactory bulb

## Abstract

Leptin, the product of the Ob(Lep) gene, is a peptide hormone that plays a major role in maintaining the balance between food intake and energy expenditure. In the brain, leptin receptors are expressed by hypothalamic cells but also in the olfactory bulb, the first central structure coding for odors, suggesting a precise function of this hormone in odor-evoked activities. Although olfaction plays a key role in feeding behavior, the ability of the olfactory bulb to integrate the energy-related signal leptin is still missing. Therefore, we studied the fate of odor-induced activity in the olfactory bulb in the genetic context of leptin deficiency using the obese ob/ob mice. By means of an odor discrimination task with concomitant local field potential recordings, we showed that ob/ob mice perform better than wild-type (WT) mice in the early stage of the task. This behavioral gain of function was associated in parallel with profound changes in neuronal oscillations in the olfactory bulb. The distribution of the peaks in the gamma frequency range was shifted toward higher frequencies in ob/ob mice compared to WT mice before learning. More notably, beta oscillatory activity, which has been shown previously to be correlated with olfactory discrimination learning, was longer and stronger in expert ob/ob mice after learning. Since oscillations in the olfactory bulb emerge from mitral to granule cell interactions, our results suggest that cellular dynamics in the olfactory bulb are deeply modified in ob/ob mice in the context of olfactory learning.

## Introduction

Dysregulation in the control of food intake is a major contributor to the rising number of obese people and strongly contributes to the obesity epidemic related lethal complications such as cardiovascular diseases and type 2 diabetes (Dietrich and Horvath, 2012). A fine tuning of feeding behavior is made possible by the central integration of peripheral energy-related circulating signals released at the periphery of the brain (Luquet and Magnan, 2009). Among these hormones, leptin is a known anorectic signal mainly expressed by the adipose tissue according to energy store (Zhang et al., 1994; Friedman and Halaas, 1998). Increasing plasma level of leptin in time of energy abundance leads to decrease in food intake and increase energy expenditure, mostly through action of leptin on hypothalamic neural substrate (Prieur et al., 2008). Disruptions in any component of the leptin signaling pathway invariably leads to hyperphagia, obesity, and corollary disease in both human and rodents (Schwartz and Porte, 2005). Likewise, the leptin-null ob/ob mice exhibit decreased energy expenditure, hyperphagia and obesity (Lindström, 2007). In addition, the obesity state *per se* is associated with the ability for leptin to properly access and regulates central neural substrate and is refereed as to “leptin resistance.” “Leptin resistance” is characterized with a state of reduced responsiveness to plasma leptin, hampering the inhibition of food intake (Zhang et al., 1994; Lee et al., 2001; Farooqi, 2006). The primary target of leptin binding in the brain is the hypothalamus. However, it was observed that neurons elsewhere in the brain (Hayes et al., 2010), including the olfactory bulb (OB), the first location in the brain to perform a spatiotemporal coding of odors, also express significant amounts of leptin receptors (Shioda et al., 1998; Prud'homme et al., 2009).

Olfactory cues are determinant for a variety of behaviors including social interactions and, importantly, for feeding behavior (Rolls, 2005; Yeomans, 2006). The OB is modulated by numerous hormones and nutrients involved in metabolic regulation (Palouzier-Paulignan et al., 2012). Both leptin receptor mRNA and immunoreactivity were found in the forebrain, in regions that massively project onto the OB, the piriform cortex and the entorhinal cortex (Elmquist et al., 1998; Shioda et al., 1998; Ur and Wilkinson, 2001) and also peripherally, in the olfactory mucosa (Baly et al., 2007). Within the OB, labeling of both long and short isoforms of the leptin receptor was observed in numerous mitral cells, in granule cells as well as on astrocytes in the glomerular and granule cells layers (Prud'homme et al., 2009). Leptin receptor immunoreactivity was found in the internal granular layer, one of the layers receiving the feedback projections. These data suggest a role for leptin in olfactory function. Indeed, in humans, a correlation has been observed between leptinemia and odor pleasantness (Trellakis et al., 2011) or odor identification (Karlsson et al., 2002). Leptin was shown to be a potent regulator of olfactory receptor neurons activity by increasing spontaneous activity and decreasing odor-evoked activity (Savigner et al., 2009). Central administration of leptin in fasted rats induces a dose-dependent decrease of olfactory detection (Julliard et al., 2007) and reduces food-odor exploration (Prud'homme et al., 2009). Accordingly, ob/ob mice detected a cracker hidden under the cage bedding faster than their wild-type littermates (Getchell et al., 2006): thus the lack of leptin seems to induce a surprising gain in olfactory function. Taken together, these data suggest that leptin influences olfactory abilities although precise mechanisms are still unknown.

Odorant molecules are detected by the olfactory receptor neurons located in the olfactory mucosa, on the caudal part of the nasal cavity (Mombaerts, 1999). Each olfactory receptor neuron (ORN) expresses one of the several hundreds of olfactory receptors (around a thousand in rodents, one third of this number in Humans) and projects to the OB. Within the olfactory glomeruli located at the surface of the OB, ORN makes excitatory synapses with the main cells of the OB, the mitral cells (Pinching and Powell, 1971). Two populations of inhibitory interneurons modulate the olfactory signal processing within the OB, the periglomerular cells located at the superficial layers and the granule cells present in deeper layers (Nagayama et al., 2014). Mitral cells project directly to neurons in the olfactory cortex (Mainland et al., 2014), mainly to the piriform cortex, which send centrifugal feedback (CF) to the OB. Olfactory cortical feedback activity tightly regulates OB interneurons activity (Boyd et al., 2012, 2015).

The olfactory system is recognized as a highly oscillatory system. Odor processing in freely moving rodents has been associated with specific modulations of the OB and the piriform cortex local field potential oscillations (LFP) (Kay, 2014; Martin and Ravel, 2014). These oscillations are also modulated by the metabolic state: oscillations elicited by food odors are enhanced in food deprived rat (Chabaud et al., 2000). Most of the time, in the context of appetitive learning, odorant sampling elicits high power oscillations in the beta (15–40 Hz) frequency band (Martin et al., 2004; Fuentes et al., 2008; Lepousez and Lledo, 2013; Chery et al., 2014). Because they underlie coincident activity, oscillations would favor temporal coordination of sensory information within brain areas and facilitation of its transfer across regions (Varela et al., 2001; Siegel et al., 2012). They are also ideally suited to subserve memory processes such as encoding, consolidation and retrieval (Engel et al., 2001; Fell and Axmacher, 2011). In addition, abnormalities of rhythmic activities in neural dynamics provide a reliable readout of neural circuit status (Cramer et al., 2012; Uhlhaas and Singer, 2015).

To date, there were no studies investigating the plasticity of olfactory behavior and related temporal dynamics in the olfactory network in the absence of leptin. We used LFP recordings in the ob/ob obese mice to study oscillatory dynamics in the OB linked to performance in an odor discrimination task. We found significant changes of neuronal oscillations in the gamma and above all in the beta frequency range suggesting that the lack of leptin induces profound changes in the interaction between the mitral and the granule cells under the influence of centrifugal inputs from the olfactory cortex.

## Materials and methods

### Animals

All protocols were approved by the Institutional Animal Care and Use Committee (Comité d'Ethique en Expérimentation Animale Paris Centre et Sud n°59) with the ethical agreement 01847.01 and carried out in compliance with EU legislation (Directive 2010/63/EU).

C57Bl/6j and ob/ob male mice were purchased from Janvier Labs (Le Genest-St-Isle, France) at the age of 6 weeks and fed with a regular diet for adult mice (A04 Diet, Safe, France). They were kept at 22 ± 0.5°C, 50 ± 5% humidity, and maintained on a 12 h light dark cycle. Animals were housed in group until the surgical procedure, and housed individually afterward. Food and water were available *ad libitum* except during the behavioral procedure when in addition to the sucrose solution received in the experimental chamber, access to water was restricted and given once a day (1 ml per mouse in their home cage at 05:00 p.m. in addition to the 0.4 mL sucrose received in the experimental chamber). Weight was measured every day during the behavioral test.

To avoid the stress of the puncture to animals involved in the learning experiment, we used a group of mice paired in age and weight to monitor glycemia. Measures were performed in ad lib condition or after 17 h-overnight fasting, by collecting a drop of blood in the tail of each mouse and using a Glucofix Monitor (Menarini Diagnotics, France).

### Electrodes implantation

Mice were implanted with a single electrode in the OB. Anesthesia was induced by 1.5–2% isoflurane, and reduced to 0.5–1% once the mouse was asleep. The level of anesthesia was confirmed by toe pinch and the absence of ocular reflex. The electrode (diameter 125 μm, stainless steel, Plastic One) was positioned stereotaxically (4.5 mm anterior, 1 mm lateral and 0.8–1.1 mm ventral relative to Bregma) at the level of the granule cells layer using electrophysiological monitoring of the signal characteristics (gamma bursts and respiratory modulation). The reference electrode was connected to a skull screw located above the posterior portion of the contralateral cortical hemisphere. The connector of the electrode was fixed on the top of the mouse's head with dental acrylic cement. A recovery period of 2 weeks followed surgical procedure before behavioral experiment.

### Electrophysiological recordings and odorant delivery

LFP signals were recorded during the entire behavioral training. Mice were connected to the recording device by a tether plugged into the implanted connector. Monopolar activity was acquired using a custom DasyLab (IOTECH, U.S.A) script driving an XCellAmp 64 amplifier (Dipsi, France) coupled with a DaqBoard 3000 USB system (IOTECH, U.S.A). Signal was sampled at 2000 Hz, amplified (x 2500) and digital filters were set at 0.1–300 Hz. Odorants were delivered using an automated perfusion system (ValveBank II, AutoMate Scientific, U.S.A) that controlled the duration and the flow rate of the stimulation (odorized air flow). A piece of filter paper, loaded with 50 μL of the odorant was used to odorize the flow. All the experiments took place in a grounded Faraday cage.

### Operant behavior

**Figure 1:**
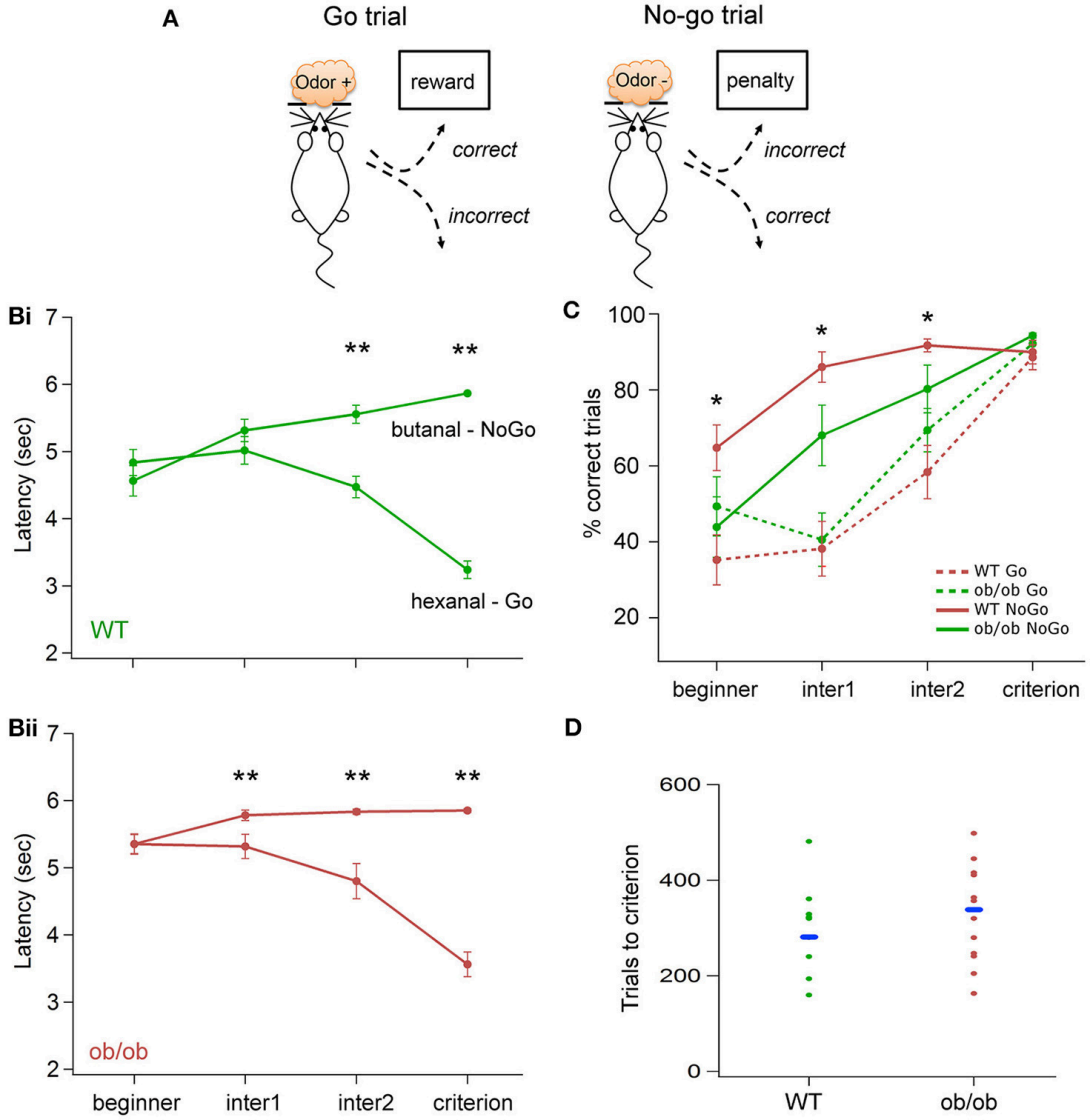
**Odor discrimination performances in WT and ob/ob mice. (A)** Schematic picturing the behavioral paradigm of the Go/No-Go task. According to the odor sampled in the odor port, a reward or a penalty was received if the animal poked into the dipper (see Materials and Methods section for details). Fifty percentage of each type of trials were delivered pseudorandomly. **(B)** Evolution of the latencies during the training for WT **(Bi)** and ob/ob **(Bii)** mice. Latency was measured for every trial as the duration between odor onset and nose poke in the dipper (correct No-Go was assigned the maximum latency of 6 s). The test was divided in 4 periods of time: *beginner*, the first 2 sessions of the Go/No-Go test, *criterion*, the 2 sessions at which mice were at the set criterion. The sessions in between were equally divided in two: *inter1* and *inter2* (see the text for details). Each value represented the average of sessions of the corresponding phase for all animals in each group (Bi: WT *n* = 14; Bii: ob/ob *n* = 10). In the ob/ob group, latency started to be significantly different for Go and No-Go trials for *inter1*, whereas significance was reached in *inter2* for the WT group (^**^p < 0.01 Mann-Whitney test). **(C)** Evolution of the percentage of correct responses for the Go and No-Go stimulations in the two groups. Performances are not significantly different between ob/ob and control mice for the Go trials but ob/ob mice have significantly better performances for the No-Go trials (^*^p < 0.05 Mann-Whitney test). **(D)** Number of trials performed by mice to reach the criterion level in the two groups. Each dot denotes one mouse. Dashes indicate the median for each group. The number of trials necessary for the mice to reach the criterion was not significantly different between ob/ob and WT animals.

Mice were trained in a Go/No-Go task based on odor discrimination (cf. Figure 1A). The experimental cage (Habitest, Coulbourn Instruments, U.S.A) was a box containing one wall equipped with two ports: (1) a self-delivery drinking system consisting of a liquid dipper and (2) a separated odor port connected to the olfactometer. Both ports were equipped with beam detectors. Clean air constantly flowed through the stimulation port. Detection of a mouse nose poke in this port triggered odorant delivery for 2 s. After odor sampling, a nose poke in the liquid dipper triggered distribution of 20 μL of sweetened water (6% sucrose in water) which remained accessible for 10 s. The whole system was controlled by Graphic State (Coulbourn Instruments, U.S.A.). The behavioral experiment was preceded by a 24 h water restriction, then the standard restriction (1 ml/mouse/day) started.

During the shaping phase, every nose poke in the odor port automatically initiated odorant delivery and triggered reward distribution. Shaping ended when the animals performed at least 20 nose pokes in one session. During the test sessions, nose poke in the odor port initiated the delivery of the odorant. Two odorants were pseudorandomly delivered for the same amount of trials per session. When animals received the odorant corresponding to Go trials they had to nose poke in the dipper to receive the reward. When it was the odorant corresponding to No-Go trials, no reward was delivered, nose poke in the dipper initiated a penalty intertrial interval of 20 s. A trial was set to 6 s including odorant delivery. A correct response was when the animal nose-poke in the dipper before the end of the trial for Go trials and when they did not nose-poke in the dipper before the end of the trial for No-Go trials. A session ended after 40 trials.

The odorant used for the shaping phase was eugenol (Sigma Aldrich, Saint-Quentin Fallavier, France) diluted at 10% in mineral oil, for the test phase, hexanal (Sigma Aldrich) diluted at 10% in mineral oil was used for Go trials, butanal (Sigma Aldrich) diluted at 15% in mineral oil was used for No-Go trials.

### Data analysis

#### Behavior

All the behavioral parameters were automatically recorded. For every trial performed by the animals, we calculated the latency between the odor onset (nose poke into the odor port) and the nose poke into the liquid dipper. For the day to day analysis, values were averaged by block of 10 consecutive trials for each odor. The percentage of correct trials was calculated for every session. Criterion performance was set at 2 consecutive days of 90% correct choice in total, including at least 80% correct choice for the No-Go trials.

#### LFP analysis

All electrophysiological data were exported and stored in a MySQL database for subsequent analysis. Data were processed using OpenElectrophy software (http://packages.python.org/OpenElectrophy; Garcia and Fourcaud-Trocmé, 2009). Data were visually inspected individually to discard trials containing artifacts.

Because the signal recorded in the OB is highly modulated in time and characterized by transient changes in frequency, we chose to use wavelet analysis (Mallat and Peyré, 2009), a particularly powerful tool for studying transient phenomena without any prior knowledge of frequency bands of interest (Tallon-Baudry and Bertrand, 1999). A continuous Morlet wavelet transform was then applied between 5 and 160 Hz and between 3 s before odor onset to 3 s after odor onset, resulting in an estimate of oscillatory power for each time and frequency values. We obtained time frequency matrix where each point represents the level of energy for a given time and a given frequency: hot color spots represent transitory oscillations.

For each map corresponding to one trial, we focused our analysis on a 1 s baseline period, before odor onset (from 2 to 1 s prior to odor onset) and a 1 s odor period, during odorant stimulation (from 0 to 1 s after odor onset). We conducted separated detection analysis for two frequency band of interest: beta (15–40 Hz) and gamma (60–150 Hz).

We first calculated LFP mean power during the baseline and odor periods in the two frequency band. We then extracted features of oscillations bursts using a method based on the wavelet ridge extraction previously described by Roux et al. (2007) and applied to biological signals by Cenier et al. (2008). Briefly, it consisted in detecting local maximum energy points above a threshold to compute the wavelet ridge, i.e., the path of lowest energy decrease. The instantaneous frequency and phase of the signal were then extracted from the ridge. To ensure that we analyze oscillations, only the bursts presenting at least 3 cycles were kept for subsequent analysis. For each trial, we extracted the number of bursts above threshold, their energy, frequency, temporal position, onset and duration.

Considering the characteristics of oscillatory activity in the OB as previously detailed (Martin and Ravel, 2014), for beta band analysis, in each trial, bursts were extracted during the odor period, with a threshold calculated at +3STD of the average value of the baseline in the same frequency range. Since gamma activity decreased during odor sampling, bursts were analyzed from the baseline with a threshold calculated from the same region of interest (baseline, 60–150 Hz). Then, the threshold was set at +2STD of the average value of the baseline to ensure the extraction of all gamma bursts.

##### Histology

Following the end of all experiments, the animals were sacrificed by a lethal dose of pentobarbital and an electrocoagulation (5 repetitions, 10 mA, 3 s) was performed through the electrode. Brain was removed and frozen. 40 μm thick coronal slices were sectioned with a cryomicrotome and Nissl staining was performed for subsequent histological examination of electrode location.

### Statistical analysis

All tests were performed comparing leptin (ob/ob) mutant mice to age- and gender-matched wild-type (WT) mice. Statistical analyses were performed using nonparametric tests. Effects of the following factors were tested: on the power, frequency and duration values, and on the number of bursts above threshold. Three independent factors were tested, the group (WT vs. ob/ob), learning (beginner vs. criterion) and the odorant (hexanal vs. butanal) using Mann-Whitney *U*-tests. One paired factor, the period factor (baseline, odor), was tested using Wilcoxon paired-samples tests. *X*^2^-test was used to test for differences in repartitions between the two groups.

## Results

We challenged ob/ob mice (*n* = 14) and matched WT mice (*n* = 18) on an odor discrimination task based on the acquisition of a Go/No-Go task (Figure 1A). During every session of the protocol, LFP was recorded in the OB of the WT and the ob/ob behaving mice. Neuronal signal recorded during the first 2 learning sessions (*beginner*) was compared to the signal recorded when mice reached the learning criterion (*criterion)*. 4 mice in each group were discarded from the analysis, either because they could not learn the operant task or because the LFP signal has been impaired.

In the beginning of the recordings, weight of ob/ob and WT were respectively 36.3 ± 1.1 and 21.2 ± 0.5 g. By the end of the recordings, the weight in both groups had slightly increased during behavior respectively to 39.9 ± 1.3 (paired *t*-test, *t* = −2.8, *p* = 0.01, *n* = 10 ob/ob) and 22.7 ± 0.7 (paired *t*-test, *t* = −2.5, *p* = 0.02, *n* = 14 WT).

We examined glycemia of ob/ob and WT mice after 17 h of fasting and found no significant difference in glycemia between the two groups (ob/ob, 1.22 ± 0.10 g/l, *n* = 5; WT 1.09 ± 0.20 g/l, *n* = 7; unpaired Student Test *t* = 1.66, *p* = 0.13).

### Odor discrimination acquisition is modified in ob/ob mice

We did not notice any trouble for the obese animals to move in the behavioral cage. They did not have difficulties to nose poke and to find the reward from 6 to 12 weeks of age. In a pilot experiment, we noticed that ob/ob mice were not motivated to move when the reward was water alone (data not shown). When we added sucrose (6% in water) and the motivation of ob/ob mice to perform the task became identical to WT mice in the context of the Go/No-Go task. We further explored whether motivational problems could happen in ob/ob vs. WT mice in response to peanut butter-flavored sugar pellets. We assessed motivation on a progressive ratio task that measures the amount of effort an animal is willing to exert to obtain food rewards. We did not observe any deficits in this operant behavior (cf. Supplementary Material). Consequently, all Go trials for all mice were rewarded with a sucrose solution.

Using a Go/No-Go task to assess odor discrimination performances gave the possibility to follow the behavior during intermediate stages of learning. We measured the latency between odor onset and the initiation of the nose-poke in the dipper (measured in seconds) as a behavioral marker. If the animal did not nose-poke, the latency was set at 6 s.

Results of behavioral performances in the Go/No-Go task showed variability between mice in the duration needed to reach the criterion (2 consecutive days of 90% correct choice in total, including at least 80% correct choice for the No-Go trials). This variability was identical for the two groups; animals needed between 4 and 12 sessions to reach the criterion. As illustrated on the Figure 1D the number of trials to criterion was not significantly different between the two groups (*p* > 0.1, Mann-Whitney's test).

We further analyzed the time course of latencies and the percentage of correct responses evolution across sessions. Each session was divided in 2 blocks of 10 trials/odor (20 trials total). To overcome the variability among individual mice, we defined 4 periods of time: *beginner* corresponded to the first 2 behavioral sessions, *criterion* corresponded to the last 2 sessions (for which the criterion has been reached). The sessions in between were split in two equal parts (half sessions are used if the total number was odd), the first half was designed as *inter1*, the second half as *inter2*. The latency has been measured for each trial of a given period, the percentage of correct response has been calculated for the entire period of time. Both have been averaged for the two odors independently.

The resulting latency curve, displayed Figure 1B revealed a difference in the early stage of learning between the ob/ob and the WT group. Indeed, the comparison between the latency after the Go and the No-Go stimulus showed a significant difference for *inter2* and *criterion* phases for both WT and ob/ob groups (*p* < 0.0005, Mann-Whitney's test). For the ob/ob group, the difference was already significant for *inter1* (*p* < 0.01, Mann-Whitney's test). Performances were plotted on Figure 1C and confirmed this effect. The percentage of correct responses for the No-Go stimulus was significantly higher for ob/ob animal since the *beginner* until *inter2* phase. In addition, no significant difference was observed for the Go stimulation, ruling out the hypothesis that ob/ob would be better at No-Go trials because they could be slower than control mice. Consequently, even if the criterion was not reached, ob/ob mice exhibited a distinct behavior for the two odors according to their value.

### The power of odor-induced beta oscillations in the OB is strongly increased in ob/ob mice

**Figure 2:**
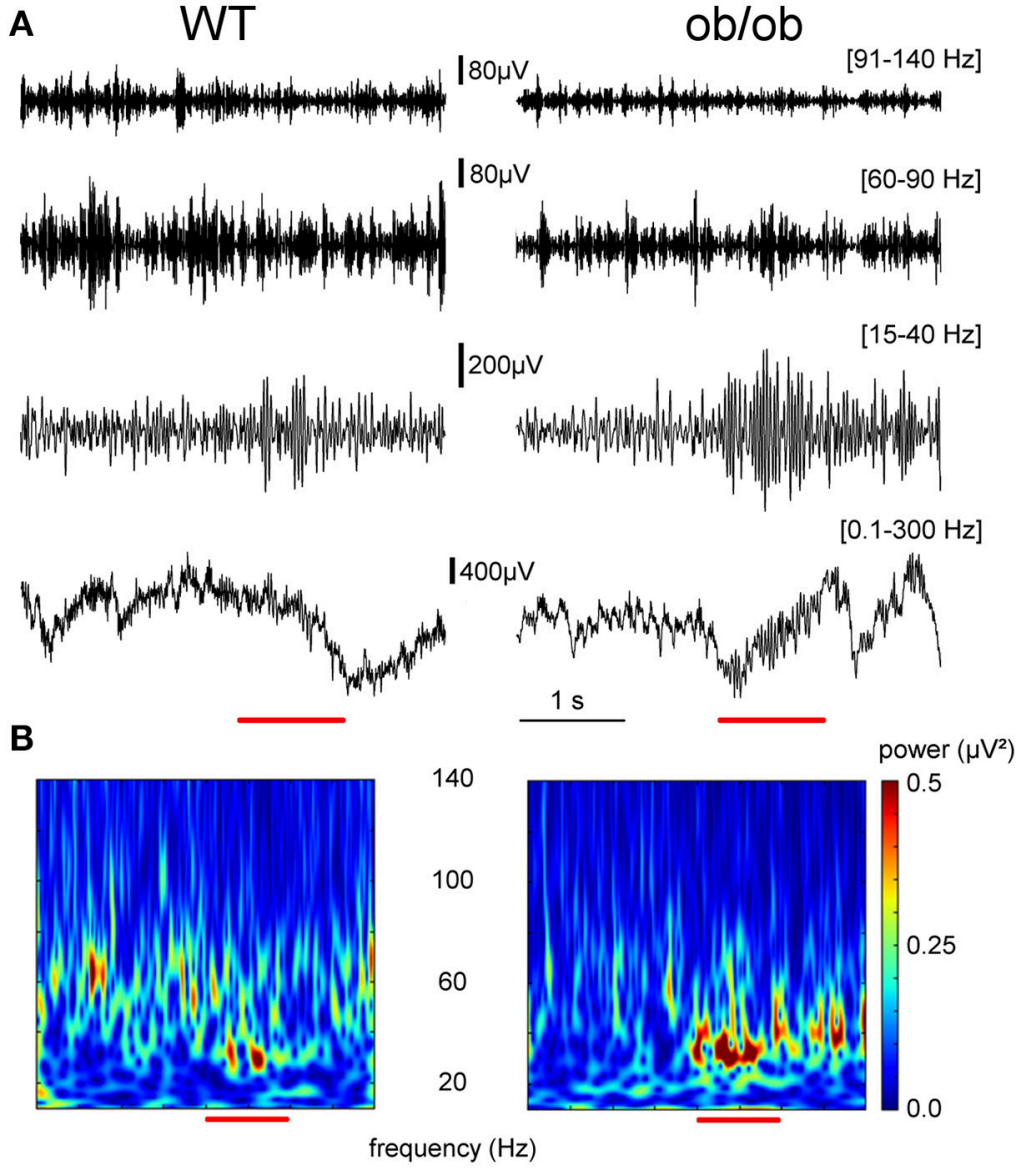
**Representative example of LFP signal recorded in WT and ob/ob mice in the OB at the criterion level. (A)** Representative example of single trials LFPs recorded in the OB during odor processing (red bar) for a WT (left) and an ob/ob mouse (right). Raw LFP (0.1–300 Hz) and the corresponding filtered signals in the frequency bands of interest in this study (beta 15–40 Hz; gamma 60–90 and 91–140 Hz). The amplitude increase in the beta band was much stronger in the ob/ob mouse compared to the WT. **(B)** Time-frequency decomposition between 5 and 140 Hz showing that odor sampling transiently changed beta oscillations (15–40 Hz). Odor was butanal. *x*-Axis, time (in s); *y*-axis, frequency from 5 to 140 Hz from bottom to top. The color scale represented signal power (μV^2^). The same scale was used for the 2 mice.

Oscillatory rhythms in the OB of awake rodents have already been described (see review in Martin and Ravel, 2014) and are characterized by a transition from high frequency gamma bursts during exploration to beta oscillation induced by odor sampling (Figures 2A,B). We focused our analysis on the characteristic of the signal when the animal received the olfactory stimulation as compared to the period preceding the nose poke. The LFP recorded in the OB of ob/ob mice displayed the typical odor-induced changes: a decrease of the gamma bursts activity (60–150 Hz) and the onset of a high power oscillation at the beta frequency (15–35 Hz).

**Figure 3:**
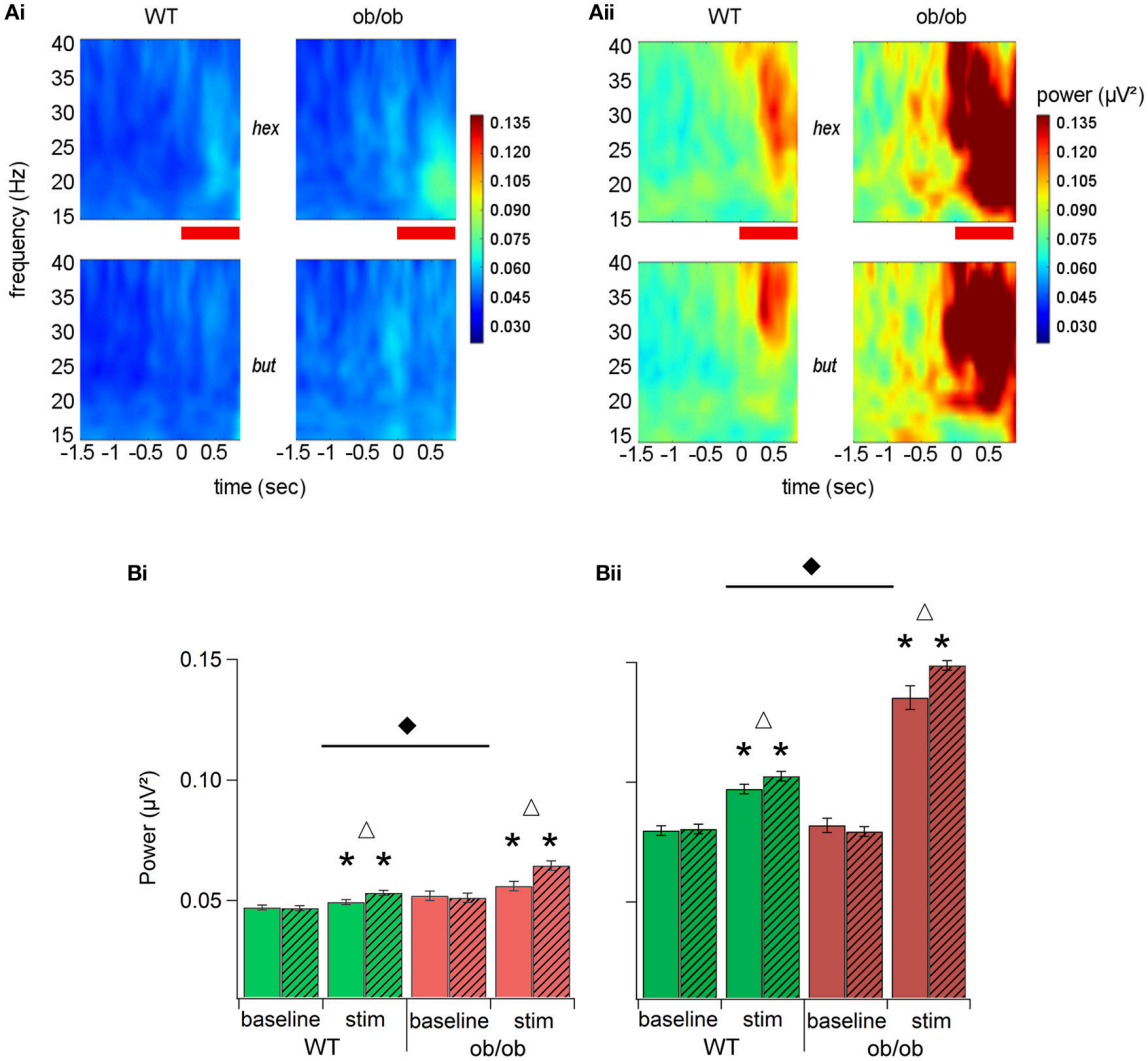
**Learning increased beta oscillatory activity in both groups but oscillations were stronger and longer in ob/ob mice. (A)** Beta band (15–40 Hz) time-frequency power plots averaged across all mice (WT *n* = 14; ob/ob *n* = 10) for beginner **(Ai)** and criterion **(Aii)** levels. The two odors (hex, hexanal: Go; but, butanal: No-Go) are represented separately as marked. Zero on the time axis is the time of the nosepoke trigger of the odor. The red horizontal bar is the odor delivery period. Color scale indicates power and is consistent for the two panels. **(B)** Mean power (±SEM) of the beta band (15–40 Hz) averaged across all mice (WT *n* = 14; ob/ob *n* = 10) for beginner **(Bi)** and criterion **(Bii)** levels for the two odorants of the pair (empty bars: hexanal; hatched bars: butanal). All mean values during odor stimulation were significantly above the mean for the baseline period (^*^Wilcoxon test *p* < 0.0001). Delta indicates significant differences between the 2 odors. Black diamond denotes a significant difference between WT and ob/ob mice.

As it can be seen in time-frequency power plots averaged across all the animals for the two groups (Figures 3Ai,ii), the overall power variation of beta oscillations in a 15–40 Hz frequency band showed a drastic difference between WT and ob/ob mice. The power of oscillations was much stronger in ob/ob mice after the task learning. Values were then averaged for two temporal windows: the odor stimulation period (from 0 to 1 s after odor onset) and the baseline (from 2 to 1 s prior to odor onset; Figures 3Bi,ii). Effect of odorant, learning level and phenotype were analyzed using a Mann-Whitney test. Power during baseline and odor periods has been analyzed as repeated measurement using a paired-sample Wilcoxon signed rank test. For the two groups, odor sampling elicited beta oscillations power increase in both beginner and criterion levels (Wilcoxon test *p* < 0.0001). There was no effect of the odorant during the baseline, in any of the conditions. An effect of the odorant during the stimulation period was only observed in beginner animals, where hexanal (go stimulus) elicited a slightly higher beta power (WT *p* < 0.0185, ob/ob *p* < 0.0005). The difference was no longer significant at criterion. For both WT and ob/ob mice, odor-induced beta oscillations power was increased by learning (WT and ob/ob: *p* < 0.0001). However, even in the first two sessions (beginner), beta power during odor sampling was much higher in ob/ob mice (*p* < 0.0001) and this difference was amplified when mice had reached the criterion (*p* < 0.0001). Beta oscillations power was enhanced by 11.3 and 3.3% for the Go and No-Go stimuli respectively in beginner ob/ob animals compared with WT; in criterion animals, beta power was 47.9 and 35.8% higher in Go and No-Go stimuli respectively in ob/ob mice compared with WT.

We noticed that beta power during the baseline time period (2–1 s prior to odor onset) was also enhanced in criterion animals compared with the beginner level (WT and ob/ob: *p* < 0.0001). However, the baseline was identical between WT and ob/ob mice in both beginner and criterion animals.

**Figure 4:**
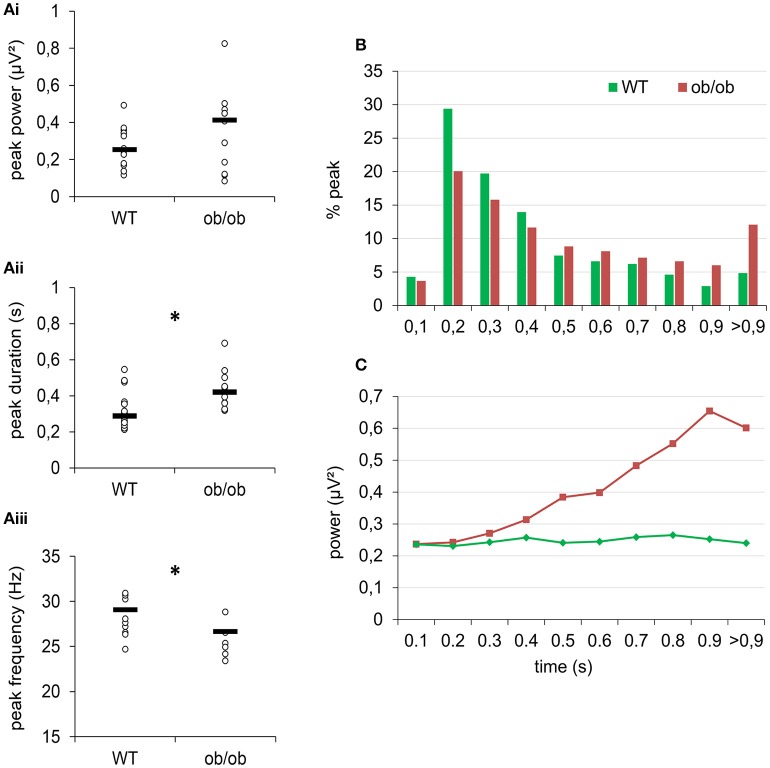
**After learning, odor sampling elicited longer and stronger oscillatory peaks in the beta band in ob/ob mice. (A)** Comparison of peaks power **(Ai)**, duration **(Aii)**, and frequency **(Aiii)** of odor-evoked beta bursts at criterion between WT and ob/ob mice. Each symbol denotes one mouse; hyphen is the median. ^*^p < 0.005 Mann-Whitney. **(B)** Distribution of the peaks of beta oscillations according to their duration for the 2 groups. For both groups, most of beta peaks lasted for 200–400 ms. However, there was an overrepresentation of long peaks (>900 ms) in ob/ob animals. **(C)** Mean power of peaks plotted as a function of beta peaks duration shows a dramatic increase of power for peaks longer than 0.5 s in ob/ob mice but not in WT. ^*^p < 0.0001 Mann-Whitney test.

The overall power increased in a time-frequency period can be explained by an increase in either the oscillatory power or the duration of beta oscillation or both. To analyze this effect thoroughly, we extracted bursts in the beta band (15–40 Hz) whose power was above a 3 STD threshold calculated from the same frequency range in the baseline period (from 2 to 1 s prior to odor onset). We then extracted the maximal power of individual significant peaks, the frequency of this maximum and the duration of individual oscillation bursts (Figure 4A). Indeed, we found that in criterion animals, the duration of beta bursts accounted for beta increase during odor sampling. As illustrated in Figure 4Aii, the duration of extracted oscillations was significantly longer in ob/ob mice compared to WT (*p* < 0.005, Mann-Whitney's test), while the difference in the maximal power of individual significant peaks did not reach significance (Figure 4Ai). We also observed that the frequency of beta bursts was decreased in ob/ob animals (*p* < 0.005; Mann-Whitney's test; Figure 4Aiii). Focusing on the duration of oscillations during odor sampling, we found that for the 2 groups of mice, a large proportion of the peaks were shorter than 400 ms. However, we noticed an increased proportion of long duration bursts in ob/ob (Figure 4B). Because the analysis was conducted between 0 and 1 s after odor onset, in both groups there was an overrepresentation of peaks >0.9 s. Additionally, whereas in WT burst power was not correlated with its duration, in ob/ob there was an impressive increase of power as the duration of the burst increased (Figure 4C). Below 400 ms duration, peaks had the same power between ob/ob and WT whereas above 500 ms, power was dramatically increased in ob/ob mice (*p* < 0.0001 Mann-Whitney's test).

To summarize, we found that LFP oscillations are stronger in the beta band (15–40 Hz) in ob/ob mice. In these animals, odorants tended to elicit longer duration burst of beta activity. In ob/ob specifically, these longer bursts reached very high energy.

### Spontaneous gamma bursts are shifted toward higher frequencies in ob/ob mice

When the animal was freely exploring the arena in the absence of odorant stimulation, LFP oscillatory activity was characterized by bursts of gamma activity superimposed on slow deflections of the signal corresponding to the respiration-related rhythm (Nguyen Chi et al., 2016). We observed that this relation to respiration was preserved in ob/ob mice.

Because of the transient nature of gamma oscillations in the OB, we extracted all the bursts whose power was above a threshold set at 2 STD of the mean power value in the time-frequency region of interest (baseline, 60–150 Hz). We then extracted the maximal power of individual significant peaks and the frequency of the maximum.

**Figure 5:**
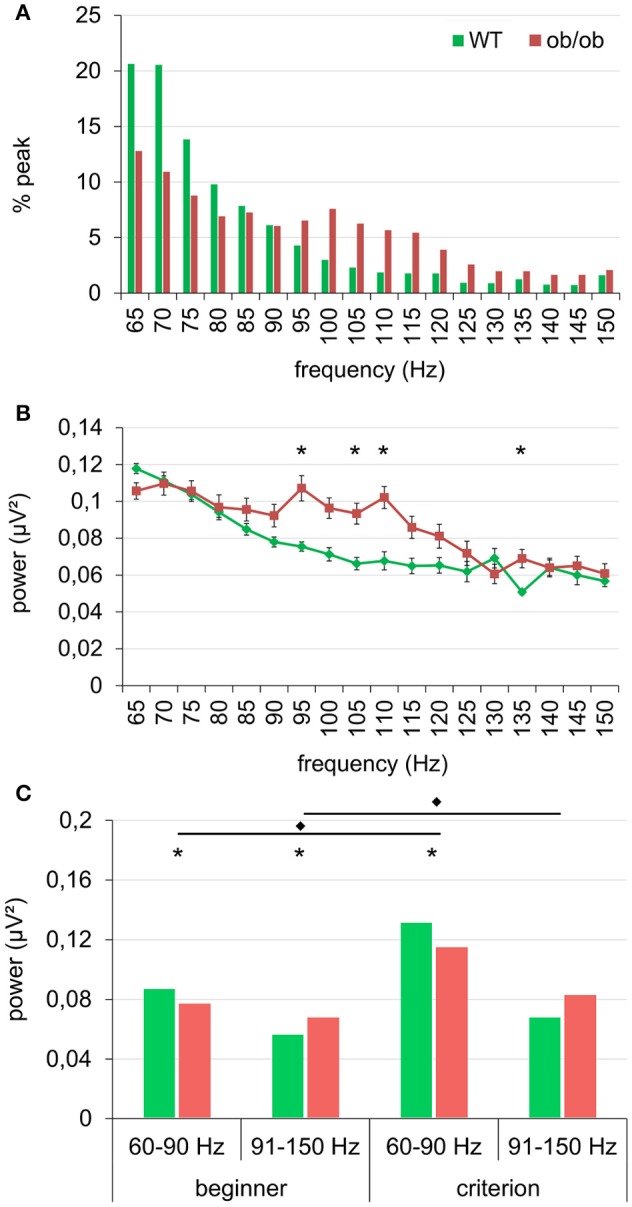
**Gamma peaks were shifted in frequency in ob/ob mice. (A)** The distribution of the peaks in the gamma frequency range became bimodal and was shifted toward higher frequencies (90–120 Hz) in ob/ob mice compared to WT mice. **(B)** This shift was also illustrated by mean power of peaks plotted as a function of their frequency (^*^p < 0.01 Mann-Whitney test). **(A,B)** are for beginner condition, similar effect was observed at the criterion. **(C)** Power of the peaks (median) in the gamma range for two frequency bands [60–90 Hz] and [91–150 Hz] confirmed the gamma shift toward higher values in ob/ob animals and denotes a general increase in gamma power after learning (^*^p < 0.01 Mann-Whitney test between WT and ob/ob; ^*^p < 0.0001 Mann-Whitney test between beginner and criterion).

In both WT and ob/ob, the median number of significant peak was 3 s^−1^. However, the analysis of the frequency and power of individual gamma bursts during baseline period revealed an alteration in ob/ob mice. The repartition of peaks frequency was changed (Test X^2^; *p* < 0.0001). As illustrated in Figure 5A in WT mice, most of the peaks had a frequency under 90 Hz. In ob/ob mice, the repartition of peaks turned bimodal with a large proportion of gamma peaks also between 90 and 115 Hz. This shift of activity was confirmed when plotting the power of peaks as a function of their frequency. The power of peaks ranging from 90 to 115 Hz was higher in ob/ob mice compared to WT mice. The difference was significant for [91–95 Hz], [101–105 Hz], [106–110 Hz], and [131–135 Hz] (*p* < 0.01, Mann-Whitney's test, Figure 5B). This difference, illustrated for the beginner session was similar after training (criterion sessions). However, results showed that gamma power during ongoing activity was enhanced by learning (Figure 5C). We further studied this effect by splitting the gamma band into two frequency bands according to the effect described above. Then we examined power of peaks ranging from [60–90 Hz] and from [91–150 Hz]. As expected from the repartition, for the 2 frequency bands and the two learning levels, gamma power was significantly different between WT and ob/ob animals (Mann-Whitney *p* < 0.0001 except for criterion level between [91–150 Hz], *p* = 0.0294). In the two frequency bands, gamma power increased after learning for both phenotypes (Mann-Whitney *p* < 0.0001).

## Discussion

Here we tested the neuronal network underlying olfactory processing using LFP recording in awake mice engaged in learning. We showed that in behaving unrestrained mice performing a Go/No-Go task for odor discrimination (i) ob/ob mice were more efficient to discriminate the two odors according to their reward in the early stages of the task (ii) OB oscillations were under the dependence of leptin availability because in ob/ob mice both beta and gamma bands, two markers of the olfactory network dynamics were modified in power and frequency.

Very few data are available on the link between leptin and olfactory performances in rodents. Intracerebroventricular injection of leptin has been shown to decrease the olfactory sensitivity in rats in the context of a conditioned olfactory aversion (Julliard et al., 2007). Consistently, when challenged in a task where they have to find a buried palatable food, ob/ob mice lacking leptin are faster in spontaneously retrieving the odorized food (Getchell et al., 2006). Our results in the odor discrimination task are in agreement with this gain of function, importantly extending it to learning: at least in the beginning of learning and because ob/ob mice have a faster transition between the beginner and the intermediate learning stage, they are quicker to discriminate between the two odors. However, in fine, ob/ob and WT mice spent the same amount of trials to reach the set criterion. Discrimination time reaction in a Go/No-Go task is determined not only by sensory processing but is also influenced by other factors such as motor behavior and motivational effects (Friedrich, 2006; Frederick et al., 2011). Despite the fact that we found no difference between ob/ob and WT mice in the context of a progressive ratio task where mice are required to press a lever to obtain a palatable reward, we cannot definitely rule out that leptin deficiency may have increased the rewarding post-ingesting effect of sucrose in the ob/ob mice (Domingos et al., 2011).

To further explore the role of leptin in OB neuronal oscillations and plasticity with learning we recorded LFP in the OB network of ob/ob compared to WT mice. In the olfactory system of awake rodents, odor sampling leads to a decrease in the gamma (60–100 Hz) bursting activity which is replaced by an increased in power of oscillations in the beta band (15–40 Hz) (Martin and Ravel, 2014). This shift in the oscillatory dynamics between gamma and beta frequencies is enhanced when odors become meaningful by associative learning and has been reported in numerous studies in the OB and piriform cortex (Martin et al., 2004; Chapuis et al., 2009; Lepousez and Lledo, 2013). In ob/ob mice, for both rewarded and non-rewarded odors, we found a very strong elevation of beta oscillation during odor sampling in addition to a shift in the frequency of gamma bursts. These specific changes in the OB temporal dynamics suggested profound modifications in the network involved in odor processing. Indeed, we have previously shown that beta power is highly dependent on CF onto the OB, most of them coming from the olfactory cortex. Pharmacological blockade of this CF abolished beta oscillation elicited by odor sampling (Martin et al., 2006). Therefore, according to previous studies (Pager et al., 1972; Chabaud et al., 2000), we hypothesize that the increase in the beta activity in ob/ob mice can be due to a modification in the CF-dependent regulation to the OB. Leptin would modulate OB physiology by acting directly or indirectly on CF.

CB1 receptors were shown to be major determinants of the OB electrical activity in fasted mice. Indeed, CB1 receptor activation selectively reduces the cortical inhibition of mitral cell spiking activity (Soria-Gómez et al., 2014). It has been suggested that leptin could act in interaction with CB1 receptors in the brain. In the hypothalamus, leptin appears to decrease food intake partly by reducing the levels of endogenous cannabinoids (Di Marzo et al., 2001). As reported for leptin receptor in ob/ob mice (Huang et al., 1997), a higher level of CB1R is found in obese Zucker rats characterized by a mutation in the leptin receptor suggesting that leptin interferes with CB1 receptor upregulation (Thanos et al., 2008). Thus, in the OB, leptin receptor activity may tune the amplitude of the oscillations and odorant processing via CB1 receptors. CB1 receptor upregulation would decrease control of CF inputs onto granule cells. This could result in changes in inhibitory tone on MC and therefore enhance oscillatory patterns. The fact that odorant-induced beta oscillations episodes were longer and stronger in ob/ob mice is in favor of an alteration in inhibitory processes. Why animals with leptin pathway dysfunction have a propensity to develop greater sensitivity to odors? Leptin having anorectic properties, it can be hypothesized that this hormone may have an inhibitory effect on the olfactory pathways necessary to search for food. The lack of the hormone—as is the case in ob/ob mice—will exacerbate challenging pathways in search of food as olfaction.

In the hypothalamus, a specific CB1-leptin interaction involving astrocytes was shown to regulate neuronal circuits and feeding (Bosier et al., 2013; Kim et al., 2014). Indeed, in the OB the presence of obR was shown on glomerular and granular astrocytes (Prud'homme et al., 2009). In addition, we have previously observed that a modification in astrocytic activity impaired oscillatory activity in the mouse OB (Martin et al., 2012). In fact, in GLAST-deficient mice, we also described a shift in gamma bursts high frequency (Martin et al., 2012). Within the OB, gamma oscillations are supported by the reciprocal synapse between mitral and granule cells (Lagier et al., 2007; David et al., 2009; Lepousez and Lledo, 2013). Taking all these facts together, specific interactions between leptin and CB1 involving astrocytes could be a key to explain changes in OB neuronal oscillations in ob/ob mice and will be the subject of future work.

In conclusion, our study demonstrated for the first time profound changes in oscillatory activities in ob/ob mice. It revealed that beside its known action as energy-related anorectic signal, leptin might directly modulate neural encoding of potent food-related cues such as odors hence potentially encoding food rewarding value (Trellakis et al., 2011). These results in ob/ob mice represent the first step in the functional study of leptin-dependent pathways within the OB and pave the way for the study of leptin as a major regulator of the OB for food odor processing in the context of feeding.

## Author contributions

Design of experiments: YC, NM, HG, and CM; data collection: YC, AE; data analysis YC, AE, HG, and CM, data interpretation: YC, SL, NM, HG, and CM writing of the paper ChM, SL, NM, HG, and CM.

## Funding

This work received funding from the IFR 144 NeuroSud-Paris and Agence Nationale de la Recherche ANR-09-JCJC-0117-01 “Neuroenergetics.” YC received a PhD funding from the French Ministry of Research (MNSER). AE is a postdoctoral researcher at FRS-FNRS, Belgium.

### Conflict of interest statement

The authors declare that the research was conducted in the absence of any commercial or financial relationships that could be construed as a potential conflict of interest.
