# Supplementary material for: Odor-Induced Neuronal Rhythms in the Olfactory Bulb Are Profoundly Modified in ob/ob Obese Mice
Source: Front Physiol. 2017 Jan 19;8:2. doi: 10.3389/fphys.2017.00002 (PMC5244437; doi:10.3389/fphys.2017.00002)
Supplement: Supplementary file 1 [file DataSheet1.DOCX]

Supplementary Material

Odor-induced neuronal rhythms in the olfactory bulb are profoundly modified in ob/ob obese mice

Yan Chelminski, Christophe Magnan, Serge Luquet, Amandine Everard, Nicolas Meunier, Hirac Gurden, Claire Martin*

*** Correspondence:** Claire Martin: [claire.martin@univ-paris-diderot.fr](mailto:claire.martin@univ-paris-diderot.fr)

# Supplementary Data: Testing the motivational effect of sugar reward in WT and ob/ob leptin-deficient mice.

Methods: Computer-controlled operant conditioning was conducted in 12 identical conditioning chambers (Phenomaster, TSE Systems GmbH, Bad Homburg, Germany) each equipped with an operant wall with a food cup in the center, surrounded by two levers located 3 cm lateral to the food cup. The left lever is designated as the active lever to obtain reward pellet delivery. To ensure responding, animals were food restricted to 90% of their initial body weight. The reinforcer was a single 20-mg peanut butter flavored sucrose tablet (TestDiet, Richmond, USA). Mice were first pre-trained to lever press for sucrose reward pellets using a fixed ratio design. Performances for the fixed ratio were not different between ob/ob and WT (Student t-test). They were then challenged on a progressive ratio task that measures the amount of effort an animal is willing to exert to obtain food rewards.


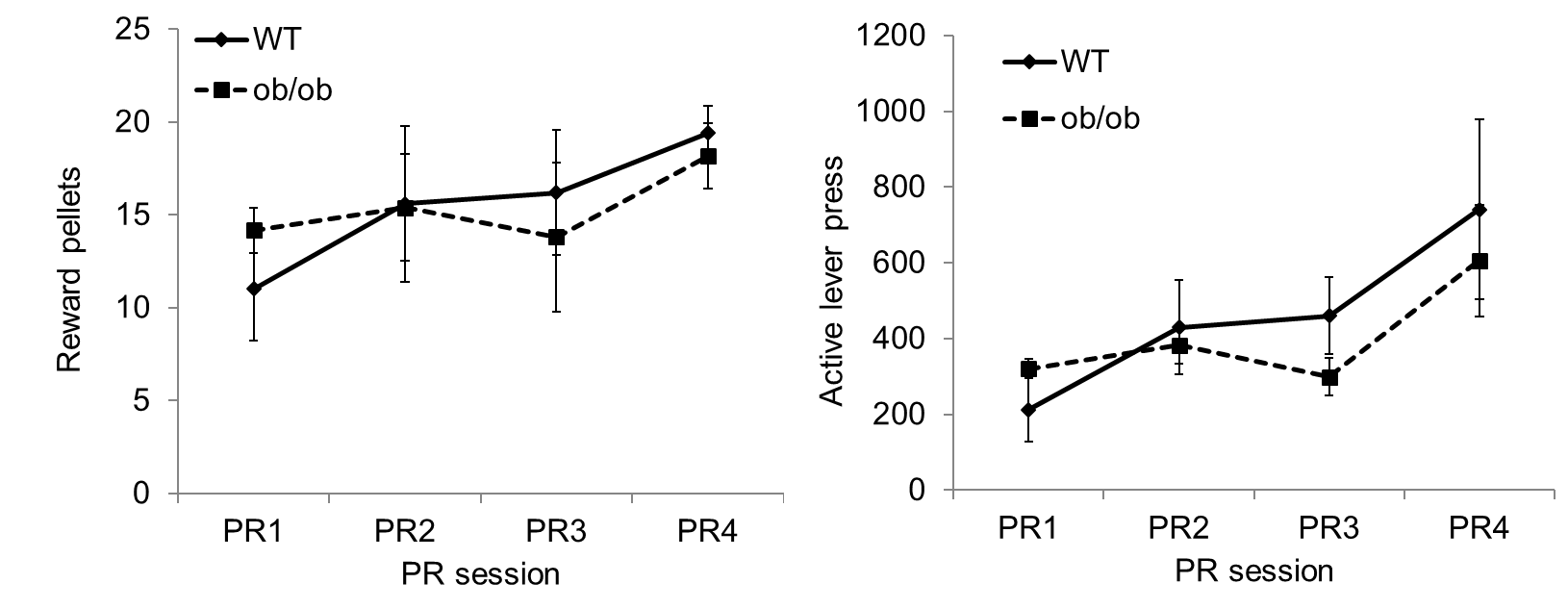


**Supplementary Figure 1.** Mean ± SEM of numbers of rewards obtained (left) and active lever presses achieved (right) were recorded during 4 sessions of the progressive ratio task. (WT n=6; ob/ob n=5). Performances for the two groups are not significantly different for the progressive ratio (Student t-test).
